# Supplementary material for: Global implementation research capacity building to address cardiovascular disease: An assessment of efforts in eight countries
Source: PLOS Glob Public Health. 2023 Sep 14;3(9):e0002237. doi: 10.1371/journal.pgph.0002237 (PMC10501667; doi:10.1371/journal.pgph.0002237)
Supplement: S1 Appendix — (DOCX) [file pgph.0002237.s001.docx]

**S1: Appendix: Capacity Building Activity Reporting Tool**

**TREIN/HyTREC Consortium**

**Capacity Building Activities**

The following table was designed to organize the different activities that each TREIN/HyTREC Study may be conducting in terms of capacity building. The Capacity Domains are based on the four dimensions of the Capacity Pyramid (Potter and Brough 2004) as incorporated into the Capacity Building Subcommittee mission statement: Tools, Skills, Staff/Infrastructure, and Structures/Systems/Roles. We have included some common activities or components of capacity building that may be a part of your study based on input from the Capacity Building Subcommittee members. Please feel free to add in additional activities that aren’t listed here. Finally, we added sections on the metrics you will be using to evaluate your study and space to describe your needs assessment (if you are conducting one) to share with the Consortium. Please complete the table to the best of your ability and send questions to [Capacity Building Subcommittee Chair] if needed. Thank you!

| **SITE** | **CAPACITY DOMAIN** | **Specific Activity/Components/Deliverables** | **Additional Details/Comment** |
| --- | --- | --- | --- |
|  | **TOOLS** | Equipment: |  |
|  |  | Computers/Digital Devices/Cell phones/printers, photocopier and projector : |  |
|  |  | Internet/Cell phone access: |  |
|  |  | IS Framework (if used): |  |
|  |  | Other: |  |
|  |  |  |  |
|  | **SKILLS** | Formal Training: |  |
|  |  | Informal Training: |  |
|  |  | Curriculum Used: |  |
|  |  | Other: Short courses and trainings |  |
|  |  |  |  |
|  | **STAFF AND INFRASTRUCTURE** | Mentors: |  |
|  |  | Investigator Time (protected): |  |
|  |  | Funding: |  |
|  |  | Other: |  |
|  |  |  |  |
|  | **Structures, Systems, Roles** | New Positions Developed: |  |
|  |  | Involvement of Ministry of Heath: |  |
|  |  | Policy Development: |  |
|  |  | Forums, Dissemination, etc: |  |
|  |  | Other: |  |
|  |  |  |  |
|  | **Metrics** | IS Process Outcomes: |  |
|  |  | Clinical Outcomes: |  |
|  |  | Other: |  |
|  |  |  |  |
|  | **Needs Assessment** | Please describe: | |
