# Supplementary material for: Global implementation research capacity building to address cardiovascular disease: An assessment of efforts in eight countries
Source: PLOS Glob Public Health. 2023 Sep 14;3(9):e0002237. doi: 10.1371/journal.pgph.0002237 (PMC10501667; doi:10.1371/journal.pgph.0002237)
Supplement: S1 Data — (PDF) [file pgph.0002237.s002.pdf]

S2: Analysis Data Set
Completed capacity-building activity reports from each site, with an updated report noting changes due to the COVID-19 pandemic.
Global Research on Implementation and Translation Science (GRIT) Consortium Member Projects are:

Project Type	Country	Project Title	LMIC Partner	US/Other HIC Partner(s)
Hy-TREC	Ghana	Uptake of task strengthening strategy for hypertension control in community health planning services in Ghana: a mixed methods study	Kwame Nkrumah University of Science and Technology<br>Kintampo Health Research Centre	Saint Louis University<br>NYU School of Medicine<br>NYU Langone Health
Hy-TREC	Guatemala	Implementing a multicomponent intervention to improve hypertension control in Central America: a cluster randomized trial in Guatemala	Institute of Nutrition of Central America and Panama (INCAP), Guatemala<br>Ministry of Health and Social Welfare of Guatemala<br>Center of Excellence for Cardiovascular Health (CESCAS), Argentina	Tulane University<br>University of Colorado
Hy-TREC	India	Integrated tracking, referral, and electronic decision support, and care coordination (I-TREC)	Centre for Chronic Disease Control<br>All India Institute of Medical Sciences	Emory University
Hy-TREC	Kenya	Strengthening Referral Networks for Management of Hypertension Across the Health System (STRENGTHS)	Moi Teaching and Referral Hospital<br>Moi University And School of Medicine<br>Academic Model Providing Access to Healthcare (AMPATH)	Purdue University College of Pharmacy
Hy-TREC	Vietnam	Conquering Hypertension in Vietnam: Solutions at Grassroots level	Health Strategy and Policy institute, Ministry of Health, Vietnam	University of Massachusetts Chan Medical School
TREIN	Malawi	NCD BRITE - Building Research capacity, Implementation and Translation Expertise for non-communicable diseases	The University of Malawi, College of Medicine<br>Ministry of Health and Population, Malawi<br>Dignitas International.<br>Partners in Health, Malawi	University of North Carolina Chapel Hill<br>London School of Hygiene and Tropical Medicine
TREIN	Nepal	Translational Research Capacity Building Initiative to Address Cardiovascular Diseases in Nepal	Dhulikhel Hospital Kathmandu University Hospital<br>Kathmandu Medical College<br>B.P. Koirala Institute of Health Sciences	University of Washington
TREIN	Rwanda	Developing T4 translational research capacity for control of hypertension in Rwanda	Ministry of Education<br>Regional Alliance for Sustainable Development	Washington University in Saint Louis.

LMIC = low to middle-income country; HIC = high income country; Hy-TREC = Hypertension Outcomes for T4 Research in Lower Middle-Income Countries; TREIN = Translation Research Capacity Building Initiative in Low Middle-Income Countries

Ghana
TREIN/HyTREC Consortium
Capacity Building Activities

The following table was designed to organize the different activities that each TREIN/HyTREC Study may be conducting in terms of capacity building. The Capacity Domains are based on the four dimensions of the Capacity Pyramid (Potter and Brough 2004) as incorporated into the Capacity Building Subcommittee mission statement: Tools, Skills, Staff/Infrastructure, and Structures/Systems/Roles. We have included some common activities or components of capacity building that may be a part of your study based on input from the Capacity Building Subcommittee members. Please feel free to add in additional activities that aren't listed here. Finally, we added sections on the metrics you will be using to evaluate your study and space to describe your needs assessment (if you are conducting one) to share with the Consortium. Please complete the table to the best of your ability and send questions to Annette Fitzpatrick (fitzpal@uw.edu) if needed. Thank you!

SITE	CAPACITY DOMAIN	Specific Activity/Components/Deliverables	Additional Details/Comment
KHRC / KNUST/ NYUSOM/ SLU	**TOOLS**	Equipment:	• Adult weighing scales<br>• Stadiometers<br>• Automatic blood pressure monitors
		Computers/Digital devices/Cell phones:	• Windows tablets for data collection
		Internet/Cell phone access:	
		IS Framework (if used):	• RE-AIM AND CFIR
		Other:	
	**SKILLS**	Formal Training:	• CHO training on study intervention and processes<br>• Training on hypertension management using ICR (Identify, Counsel, and Refer)<br>• Training on general knowledge of hypertension<br>• Training on proper measuring of Blood pressure<br>• Training of Coaches on task strengthening facilitation<br>• Training of Health Management Team to provide supportive supervision.
		Informal Training:	• Coordinators site visit and interaction<br>• Regular telephone conversations between Coordinators, Coaches and CHOs<br>• WhatsApp learning environment for engaging CHOs and Coaches
		Curriculum Used:	• Uptake TASSH training manual (designed based on Ghana's NCD policy and standard treatment guidelines)
		Other:	
	**STAFF AND INFRASTRUCTURE**	Mentors:	
		Investigator Time (protected):	• Dr(s). Plange-Rhule, Ogedegbe, Asante, and Iwelunmor

		Funding:	• NIH / NHLBI
		Other:	
	**Structures, Systems, Roles**	New Positions Developed:	None
		Involvement of Ministry of Heath:	• Strategic Partners from the Ghana Health services and Ministry of Health form a major part of the study steering committee both at the national and regional levels<br>• Various presentations on the study have been made to the various forums of the Ghana Health services.<br>• The Ghana Health services has also granted IRB approval for the study
		Policy Development:	
		Forums, Dissemination, etc:	• Community and Opinion Leaders Engagements<br>• Policy briefs<br>• Publications<br>• Progress presentations at the various review meetings of the Ghana Health Service (National, Regional and Districts)<br>• Periodic Progress Report submitted to strategic partners
		Other:	
	**Metrics**	IS Process Outcomes:	• Rate of adoption of TASSH at 12 months post randomization; Sustainability of TASSH at 24 months post randomization at the participating CHPS zones
		Clinical Outcomes:	• Mean reduction in systolic blood pressure (BP) at 12 months
		Other:	• Mediators (implementation climate, leadership support, organizational capacity and provider- level characteristics) of the uptake of TASSH across the CHPS zones at 12 and 24 months.
	**Needs Assessment**	Please describe:<br>Practice capacity survey was conducted to assess the readiness of the health workers and facilities in partaking in the study. The survey was guided by the consolidated framework for implementation research (CFIR). 180 responses were received from health workers in six contiguous districts of the study region in Brong Ahafo. Community health workers demonstrated sufficient knowledge of lifestyle modification for hypertension management. Their knowledge was however limited in the BP threshold for initiating treatment. The health workers also reported receiving very minimum prior training on hypertension screening and management.<br><br>Regular engagement of community health management teams.	

Ghana COVID-19 Update
TREIN/HyTREC Consortium
Capacity Building Activities

Previously, you shared the below information regarding your capacity building activities. The Capacity Building Committee is summarizing this data in a manuscript; in order to ensure we have the most up-to-date information; we need your help.

Please review the table and: (1) update any incorrect/incomplete information and (2) indicate any changes or adaptations to these activities (e.g., delay in activity, changes in delivery mode, inability to complete) due to the COVID-19 pandemic. Please complete the table to the best of your ability and send questions to Mary Beth Weber (mbweber@emory.edu) if needed. This activity should take no more than 30 minutes to complete. We appreciate your assistance.

CAPACITY DOMAIN	Specific Activity/Components/ Deliverables	Additional Details/Comment	Changes/Adaptations due to COVID-19
**TOOLS**	Equipment:	• Adult weighing scales<br>• Stadiometers<br>• Automatic blood pressure monitors	NONE
	Computers/Digital devices/Cell phones:	• Windows tablets for data collection	NONE
	Internet/Cell phone access:		NONE
	IS Framework (if used):	• RE-AIM AND CFIR	NONE
	Other:		NONE
**SKILLS**	Formal Training:	• CHO training on study intervention and processes<br>• Training on hypertension management using ICR (Identify, Counsel, and Refer)<br>• Training on general knowledge of hypertension<br>• Training on proper measuring of Blood pressure<br>• Training of Coaches on task strengthening facilitation<br>• Training of Health Management Team to provide supportive supervision.	TRAINING WAS DONE IN BATCHES, OVER A PERIOD OF TWO WEEKS INSTEAD OF THE USUAL THREE DAYS.<br><br>HEALTH MANAGEMENT TRAINING HAS DELAYED BECAUSE THE PERSONNEL ARE ENGAGED IN COVID EMERGENCIES

	Informal Training:	• Coordinators site visit and interaction<br>• Regular telephone conversations between Coordinators, Coaches and CHOs<br>• WhatsApp learning environment for engaging CHOs and Coaches	ALL ACTIVITIES ARE CURRENTLY ONGOING
	Curriculum Used:	• Uptake TASSH training manual (designed based on Ghana's NCD policy and standard treatment guidelines)	NONE
	Other:		
**STAFF AND INFRASTRUCTURE**	Mentors:		
	Investigator Time (protected):	• Dr(s). Plange-Rhule, Ogedegbe, Asante, and Iwelunmor	PROF PLANGE-RHULE PASSED DUE TO COVID COMPLICATIONS. DR. ASANTE IS THE NEW PI. THE KNUST TEAM IS NOW LED BY DR. KWEKU BEDU-ADDO
	Funding:	• NIH / NHLBI	
	Other:		
**Structures, Systems, Roles**	New Positions Developed:	None	NONE
	Involvement of Ministry of Heath:	• Strategic Partners from the Ghana Health services and Ministry of Health form a major part of the study steering committee both at the national, regional and district levels<br>• Various presentations on the study have been made to these stakeholders at the Ghana Health services and Ministry of Health.<br>• The Ghana Health services Ethics Review Board has also granted IRB approval for the study	THE MINISTRY IS STILL INVOLVED WITH THE STUDY. THE STEERING COMMITTEE MEETING FOR 2021 IS YET TO BE HELD.

	Policy Development:	At the stakeholder dialogue meetings various policy recommendation have been proffered to the Ghana Health Service to strengthen the peripheral facility in terms of management of Hypertension. Also, the various stakeholders have also suggested strategies for the successful implementation and sustainability of this study.	
	Forums, Dissemination, etc:	• Community and Opinion Leaders Engagements<br>• Policy briefs<br>• Publications<br>• Progress presentations at the various review meetings of the Ghana Health Service (Districts, Regional and National)<br>• Periodic Progress Report submitted to strategic partners.	
	Other:		
**Metrics**	IS Process Outcomes:	• Rate of adoption of TASSH at 12 months post randomization; Sustainability of TASSH at 24 months post randomization at the participating CHPS zones	NONE
	Clinical Outcomes:	• Mean reduction in systolic blood pressure (BP) at 12 months	NONE
	Other:	• Mediators (implementation climate, leadership support, organizational capacity and provider- level characteristics) of the uptake of TASSH across the CHPS zones at 12 and 24 months.	NONE

**Needs Assessment**	Please describe:<br>Practice capacity survey was conducted to assess the readiness of the health workers and facilities in partaking in the study. The survey was guided by the consolidated framework for implementation research (CFIR). 180 responses were received from health workers in six contiguous districts of the study region in Brong Ahafo. Community health workers demonstrated sufficient knowledge of lifestyle modification for hypertension management. Their knowledge was however limited in the BP threshold for initiating treatment. The health workers also reported receiving very minimum prior training on hypertension screening and management.<br><br>Regular engagement of community health management teams.	
**Additional Comments/Feedback on COVID-19 Impact Not Listed Above:**		

Guatemala
TREIN/HyTREC Consortium
Capacity Building Activities

The following table was designed to organize the different activities that each TREIN/HyTREC Study may be conducting in terms of capacity building. The Capacity Domains are based on the four dimensions of the Capacity Pyramid (Potter and Brough 2004) as incorporated into the Capacity Building Subcommittee mission statement: Tools, Skills, Staff/Infrastructure, and Structures/Systems/Roles. We have included some common activities or components of capacity building that may be a part of your study based on input from the Capacity Building Subcommittee members. Please feel free to add in additional activities that aren't listed here. Finally, we added sections on the metrics you will be using to evaluate your study and space to describe your needs assessment (if you are conducting one) to share with the Consortium. Please complete the table to the best of your ability and send questions to Annette Fitzpatrick (fitzpal@uw.edu) if needed. Thank you!

**CAPACITY DOMAIN**	**Specific Activity/Components/Deliverables**	
	**Research Capacity Building**	**Health System Capacity Building**
**TOOLS**	**Equipment**:<br>BP monitors, OMRON 907XL	BP monitors, OMRON HEM7121<br>Weight Balances<br>Stadiometers
	**Computers/Digital devices/Cell phones:**<br><br>Tablets for data collection<br>Redcap<br>NVivo<br>STATA	
	**Internet/Cell phone access:**<br><br>Zoom<br><br>Internet access for communication among research assistants and research coordinator via Whatsapp	
	**IS Framework (if used):**<br><br>RE-AIM	
	**Other**:<br><br>ASANA Software to coordinate research assistants and data collectors<br><br>Data gathering forms, lists of patients with hypertension, checklists	Data gathering forms, lists of patients with hypertension, checklists

**SKILLS**	**Formal Training:**<br>Not applicable	Not applicable.
	**Informal Training:**<br><br>Clinical research competencies for research assistants<br><br>Basic clinical research competencies for data collectors (data collection)<br><br>Training based on WHO clinical research competencies framework<br><br>Participation in Webinars about Implementation Science<br><br>Participation of Research Coordinator (junior investigator) in HY TREC / TREIN consortium international meetings.	Healthcare provider certification supported by national Department of Training (DECAP – Departamento de Capacitación) and co-created with the Unit for Promotion and Education for Health (PROEDUSA):<br><br>2 day training for physicians, professional nurses, auxiliary nurses on standardized hypertension treatment (pharmacologic, non-pharmacologic) and team-based collaborative care<br><br>Field certification on health Coaching for auxiliary nurses
	**Curriculum Used:**<br>Not applicable	Training based on an updated version of the previously validated manual "Corazon Sano y Feliz"
	**Other**:	
**STAFF AND INFRASTRUCTURE**	**Mentors**:<br>Manuel Ramirez, MD PhD<br>Jiang He, MD, PhD<br>Vilma Irazola, MD PhD MPH<br>Meredith Fort, PhD	
	Investigator Time (protected):<br><br>Study team's time dedicated to the HyTREC project	
	**Funding**:<br><br>NHLBI	
	**Other**:	
	**New Positions Developed**	

**Structures, Systems, Roles**	1 Lead Research coordinator<br>2 Research assistants<br>18 data collectors	
	**Involvement of Ministry of Heath:**	Ministry of Health provider teams will implement the intervention.<br><br>Representatives of the Ministry of Health's Comprehensive System for Healthcare (Sistema Integral de Atención en Salud, SIAS), Unit of supervision and monitoring (USME), Financial unit and PROEDUSA.<br><br>Chronic Disease Program Director.
	**Policy Development:**	Dissemination of Guatemalan guidelines related to hypertension and cardiovascular care
	**Forums, Dissemination, etc:**	Multiple meetings with central-level managers at Ministry of Health, Health area (state level) administrators<br><br>Trainings for healthcare providers
	**Other:**	
**Metrics**	IS Process Outcomes:<br><br>Study will evaluate all dimensions of RE AIM. We apply RE-AIM at multiple levels: patients, providers, health system (district, department & central levels).<br><br>Specific outcomes: acceptability, adoption, reach, appropriateness, feasibility, fidelity, and sustainability.<br><br>Cost-effectiveness analysis.	
	Clinical Outcomes (effectiveness):<br>Primary outcome: % patients with controlled BP<br><br><u>Other patient-level effectiveness measures:</u><br>Adherence to medications<br>Lifestyle: DASH diet, physical activity, tobacco and alcohol consumption<br>Body Mass Index<br>Quality of life<br>Stage of change	

	Other:	
**Needs Assessment**	We conducted a qualitative participatory needs assessment to adapt the program, previously implemented in Argentina, to the Guatemalan context. The needs assessment evaluated the six WHO Building Blocks of a Health System in relation to the facilitators, barriers and knowledge gaps of hypertension care in the country. The study was multilevel, as we conducted semi-structured interviews and focus groups with participants from all levels of the National Public Health System (Central-level Ministry of Health administrators and health directors, physicians, nurses, auxiliary nurses, hypertensive patients, relatives, traditional healers and midwives).<br><br>Upon finalizing the needs assessment data collection, we conducted adaptation workshops, to disseminate the information obtained during the needs assessment and adapt the intervention to the Guatemalan context. We conducted workshops in all 5 departments (states) where the study will be implemented, at the department-level and at the district-level. In addition, we conducted multiple workshops with doctors, administrators and program directors in the Comprehensive System for Healthcare (Sistema Integral de Atención en Salud, SIAS), at the Ministry of Health's central level.<br><br>Assessment at the facility level of each site that will be included in the study. This assessment provides detailed existing resources at each of the facilities. The information informs the needs assessment and will help contextualize the study.	

Guatemala
TREIN/HyTREC Consortium
Capacity Building Activities

Previously, you shared the below information regarding your capacity building activities. The Capacity Building Committee is summarizing this data in a manuscript; in order to ensure we have the most up-to-date information, we need your help.

Please review the table and: (1) update any incorrect/incomplete information and (2) indicate any changes or adaptations to these activities (e.g., delay in activity, changes in delivery mode, inability to complete) due to the COVID-19 pandemic. Please complete the table to the best of your ability and send questions to Mary Beth Weber (mbweber@emory.edu) if needed. This activity should take no more than 30 minutes to complete. We appreciate your assistance.

**CAPACITY DOMAIN**	**Specific Activity/Components/Deliverables**		**Changes/Adaptations due to COVID-19**
	**Research Capacity Building**	**Health System Capacity Building**	
**TOOLS**	**Equipment**:<br>BP monitors, OMRON 907XL	BP monitors, OMRON HEM7121<br>Weight Balances<br>Stadiometers	
	**Computers/Digital devices/Cell phones:**<br><br>Tablets for data collection<br>Redcap<br>NVivo<br>STATA		A telephone call system was used so that field evaluators could monitor the situation of the participants, especially focusing on the availability and delivery of antihypertensive medications.
	**Internet/Cell phone access:**<br><br>Zoom<br><br>Internet access for communication among research assistants and research coordinator via Whatsapp		
	**IS Framework (if used):**<br><br>RE-AIM		
	**Other**:<br><br>ASANA Software to coordinate research assistants and data collectors	Data gathering forms, lists of patients with hypertension, checklists	

	Data gathering forms, lists of patients with hypertension, checklists		
**SKILLS**	**Formal Training:**<br>Not applicable	Not applicable.	
	**Informal Training:**<br><br>Clinical research competencies for research assistants<br><br>Basic clinical research competencies for data collectors (data collection)<br><br>Training based on WHO clinical research competencies framework<br><br>Participation in Webinars about Implementation Science<br><br>Participation of Research Coordinator (junior investigator) in HY TREC / TREIN consortium international meetings.	Healthcare provider certification supported by national Department of Training (DECAP – Departamento de Capacitación) and co-created with the Unit for Promotion and Education for Health (PROEDUSA):<br><br>2 day training for physicians, professional nurses, auxiliary nurses on standardized hypertension treatment (pharmacologic, non-pharmacologic) and team-based collaborative care<br><br>Field certification on health Coaching for auxiliary nurses	For new providers who entered the study during the COVID-19 pandemic, the original training was modified and adapted to 1 day of face-to-face training and remote support with theoretical and educational materials via WhatsApp or phone call.
	**Curriculum Used:**<br>Not applicable	Training based on an updated version of the previously validated manual "Corazon Sano y Feliz"	
	**Other**:		
**STAFF AND INFRASTRUCTURE**	**Mentors**:<br>Manuel Ramirez, MD PhD<br>Jiang He, MD, PhD<br>Vilma Irazola, MD PhD MPH<br>Meredith Fort, PhD		
	Investigator Time (protected):<br><br>Study team's time dedicated to the HyTREC project		
	**Funding**:<br><br>NHLBI		

	**Other**:		
**Structures, Systems, Roles**	**New Positions Developed**<br><br>1 Lead Research coordinator<br>2 Research assistants<br>18 data collectors		
	**Involvement of Ministry of Heath:**	Ministry of Health provider teams will implement the intervention.<br><br>Representatives of the Ministry of Health's Comprehensive System for Healthcare (Sistema Integral de Atención en Salud, SIAS), Unit of supervision and monitoring (USME), Financial unit and PROEDUSA.<br><br>Chronic Disease Program Director.	Support was required from the health area directors and leaders of the health districts of the MoH in order to carry out the intervention activities in all districts.
	**Policy Development:**	Dissemination of Guatemalan guidelines related to hypertension and cardiovascular care	
	**Forums, Dissemination, etc:**	Multiple meetings with central-level managers at Ministry of Health, Health area (state level) administrators<br><br>Trainings for healthcare providers	
	**Other:**		
**Metrics**	IS Process Outcomes:<br><br>Study will evaluate all dimensions of RE AIM. We apply RE-AIM at multiple levels: patients, providers, health system (district, department & central levels).<br><br>Specific outcomes: acceptability, adoption, reach, appropriateness, feasibility, fidelity, and sustainability.<br><br>Cost-effectiveness analysis.		

	Clinical Outcomes (effectiveness):<br>Primary outcome: % patients with controlled BP<br><br><u>Other patient-level effectiveness measures:</u><br>Adherence to medications<br>Lifestyle: DASH diet, physical activity, tobacco and alcohol consumption<br>Body Mass Index<br>Quality of life<br>Stage of change		Outcome assessments at 6-month follow-up visits were mostly affected. Most of them were done but with a considerable delay of 3-5 months.
	Other:		
**Needs Assessment**	We conducted a qualitative participatory needs assessment to adapt the program, previously implemented in Argentina, to the Guatemalan context. The needs assessment evaluated the six WHO Building Blocks of a Health System in relation to the facilitators, barriers and knowledge gaps of hypertension care in the country. The study was multilevel, as we conducted semi-structured interviews and focus groups with participants from all levels of the National Public Health System (Central-level Ministry of Health administrators and health directors, physicians, nurses, auxiliary nurses, hypertensive patients, relatives, traditional healers and midwives).<br><br>Upon finalizing the needs assessment data collection, we conducted adaptation workshops, to disseminate the information obtained during the needs assessment and adapt the intervention to the Guatemalan context. We conducted workshops in all 5 departments (states) where the study will be implemented, at the department-level and at the district-level. In addition, we conducted multiple workshops with doctors, administrators and program directors in the Comprehensive System for Healthcare (Sistema Integral de Atención en Salud, SIAS), at the Ministry of Health's central level.<br><br>Assessment at the facility level of each site that will be included in the study. This assessment provides detailed existing resources at each of the facilities. The information informs the needs assessment and will help contextualize the study.		
**Additional Comments/Feedback on COVID-19 Impact Not Listed Above:**			

India
TREIN/HyTREC Consortiumnd
Capacity Building Activities

The following table was designed to organize the different activities that each TREIN/HyTREC Study may be conducting in terms of capacity building. The Capacity Domains are based on the four dimensions of the Capacity Pyramid (Potter and Brough 2004) as incorporated into the Capacity Building Subcommittee mission statement: Tools, Skills, Staff/Infrastructure, and Structures/Systems/Roles. We have included some common activities or components of capacity building that may be a part of your study based on input from the Capacity Building Subcommittee members. Please feel free to add in additional activities that aren't listed here. Finally, we added sections on the metrics you will be using to evaluate your study and space to describe your needs assessment (if you are conducting one) to share with the Consortium. Please complete the table to the best of your ability and send questions to Annette Fitzpatrick (fitzpal@uw.edu) if needed. Thank you!

SITE	CAPACITY DOMAIN	Specific Activity/ Components/ Deliverables	Additional Details/Comment
	TOOLS	Equipment:	• Digital Bp instruments<br>• Digital Weighing scale<br>• Portable stadiometers<br>• Measuring tape/Girth meter<br>• Glucometer and glucometer strips<br>• Gloves<br>• Alcohol swabs<br>• Carry bag
		Computers/Digital devices/Cell phones:	• Laptops are provided to Project Manager, Project Coordinator and Supervisors for reporting, preparing power points<br>• Laptop also provided to Data manager for data cleaning and management<br>• Field workers are given tablets for filling the electronic version of the baseline survey<br>• Commcare application from Dimagi- Paid application for baseline data collection
		Internet/Cell phone access:	4G sim are provided to field team for syncing of data
		IS Framework (if used):	RE-AIM
		Other:	
	SKILLS	Formal Training:	❖ **Formal training of Filed workers on filling baseline survey**<br><br>Formal training and retraining of Field Workers have been conducted on following aspects<br>• 5 day training and 3 day Retraining was conducted

			• Knowledge about the roles and responsibilities of Field Workers<br>• Household listing using Google Maps<br>• Method to conduct baseline survey<br>• Method to record anthropometric measurements<br>• Technical aspect and troubleshooting of Tablets<br><br>❖ **Formal training of Field Workers on conducting facility Assessment**<br><br>Training on the tool was given about how to record responses<br><br>❖ **Formal Training of Supervisors to conduct Geocoding using Garmin Devices**<br><br>❖ **Formal Training of Data Manager on building Commcare application and Data management on server**<br><br>❖ **Training of providers on IT platform**
		Informal Training:	Hand holding exercises for Field workers was done in initial weeks to build confidence
		Curriculum Used:	• Curriculum was designed based on the trainings that are conducted in other similar projects<br>• Training manuals were prepared for the project which was given to all the team members for quick reference<br>• A formal timeline pertaining to each activity is also prepared that is referred on weekly basis
		Other:	
	**STAFF AND INFRASTRUCTURE**	Mentors:	Dr Nikhil Tandon (PI)<br>Dr Shivani Patel (CO– I)<br>Dr. Prabhaker Dorairaj (CO-I)<br>Dr Sailesh Mohan (CO– I)<br>Dr. Venkat Narayan (CO-I)<br>Dr. Mary Beth Weber (CO-I)<br>Dr. Mohammed K. Ali (CO-I)
		Investigator Time (protected):	• One hour Operation group meeting is held every Wednesday which is attended by operations group including PI and Co- Investigators to discuss weekly update<br>• One hour Data call every Tuesday to discuss data issues, progress of data collection, data management, analysis<br>• A one day bi- annual Investigators meeting to discuss about project updates, action items<br>• Six monthly site visits by Investigators
		Funding:	NHLBI

		Other:	
	**Structures, Systems, Roles**	New Positions Developed:	We have leveraged several senior research associates affiliated with our partner institutions to assist in the capacity building activities. To date, these are unfunded positions.
		Involvement of Ministry of Heath:	• Study team is working in close collaboration with Department of Health and Family Welfare, Punjab State and Ministry Health And Family Welfare, India (NCD Division) to implement project in Punjab<br>• Letter of Support from Ministry Health And Family Welfare, India (NCD Division) and Letter of Support from Civil Surgeon to facilitate data collection across intervention and control blocks<br>• Training of nurses and medical officers will occur in this calendar year.
		Policy Development:	Dr Nikhil Tandon is a part of Expert group that is advising Policy makers in Ministry to Develop electronic case record form and Clinical decision Support System in the National program that is being rolled out Pan India for screening and Management of NCDs (*National Program For Control And Prevention Of Cancers, Diabetes, CVD And Stroke*.)
		Forums, Dissemination, etc:	We plan to conduct a dissemination meeting.
		Other:	
	**Metrics**	IS Process Outcomes:	Guided by the RE-AIM framework, we will evaluate program **R**each (e.g., %'s screened in the community, tracked over time, and referred), **E**ffectiveness (e.g., % with controlled hypertension in the community), **A**doption (e.g., % of facilities using the I-TREC; provider reception to I-TREC), **I**mplementation outcomes (e.g., concordance between facility paper registry I-TREC database; routine operational costs per patient), and **M**aintenance (e.g., % of facilities utilizing I-TREC 6 months after the active intervention). Data will come from community-surveys, patient focus group discussions, provider interviews, and the I-TREC platform.
		Clinical Outcomes:	Data points recorded in the I-TREC system will be analyzed by month to examine trends over time and seasonality. We will also evaluate change patient outcomes over time (e.g., mean SBP change). While several of the I-TREC platform indicators are purely descriptive measures of performance (e.g., mean time for data upload), other indicators of healthcare delivery may be compared between I-TREC facilities and comparison group facilities (e.g., patient volume).
		Other:	

	**Needs Assessment**	• Community epidemiological survey<br>• Qualitative interviews with community members and providers<br>• Facility assessments

India
TREIN/HyTREC Consortium
Capacity Building Activities

Previously, you shared the below information regarding your capacity building activities. The Capacity Building Committee is summarizing this data in a manuscript; in order to ensure we have the most up-to-date information, we need your help.

Please review the table and: (1) update any incorrect/incomplete information and (2) indicate any changes or adaptations to these activities (e.g., delay in activity, changes in delivery mode, inability to complete) due to the COVID-19 pandemic. Please complete the table to the best of your ability and send questions to Mary Beth Weber (mbweber@emory.edu) if needed. This activity should take no more than 30 minutes to complete. We appreciate your assistance.

CAPACITY DOMAIN	Specific Activity/ Components/ Deliverables	Additional Details/Comment	Changes/Adaptations due to COVID-19
**TOOLS**	Equipment:	• Digital Bp instruments<br>• Digital Weighing scale<br>• Portable stadiometers<br>• Measuring tape/Girth meter<br>• Glucometer and glucometer strips<br>• Gloves<br>• Alcohol swabs<br>• Carry bag	
	Computers/Digital devices/Cell phones:	• Laptops are provided to Project Manager, Project Coordinator and Supervisors for reporting, preparing power points<br>• Laptop also provided to Data manager for data cleaning and management<br>• Field workers are given tablets for filling the electronic version of the baseline survey<br>• Commcare application from Dimagi- Paid application for baseline data collection	
	Internet/Cell phone access:	4G sim are provided to field team for syncing of data	
	IS Framework (if used):	RE-AIM	
	Other:		

**SKILLS**	Formal Training:	❖ **Formal training of Filed workers on filling baseline survey**<br><br>Formal training and retraining of Field Workers have been conducted on following aspects<br>• 5 day training and 3 day Retraining was conducted<br>• Knowledge about the roles and responsibilities of Field Workers<br>• Household listing using Google Maps<br>• Method to conduct baseline survey<br>• Method to record anthropometric measurements<br>• Technical aspect and troubleshooting of Tablets<br><br>❖ **Formal training of Field Workers on conducting facility Assessment**<br><br>Training on the tool was given about how to record responses<br><br>❖ **Formal Training of Supervisors to conduct Geocoding using Garmin Devices**<br><br>❖ **Formal Training of Data Manager on building Commcare application and Data management on server**<br><br>❖ **Training of providers on IT platform**	To accommodate social distancing policies, several training activities were moved to zoom and/or small group in-person sessions. Therefore, training took more days and manpower to implement.
	Informal Training:	Hand holding exercises for Field workers was done in initial weeks to build confidence	
	Curriculum Used:	• Curriculum was designed based on the trainings that are conducted in other similar projects<br>• Training manuals were prepared for the project which was given to all the team members for quick reference<br>• A formal timeline pertaining to each activity is also prepared that is referred on weekly basis	
	Other:		

**STAFF AND INFRASTRUCTURE**	Mentors:	Dr Nikhil Tandon (PI)<br>Dr Shivani Patel (CO– I)<br>Dr. Prabhaker Dorairaj (CO-I)<br>Dr Sailesh Mohan (CO– I)<br>Dr. Venkat Narayan (CO-I)<br>Dr. Mary Beth Weber (CO-I)<br>Dr. Mohammed K. Ali (CO-I)	
	Investigator Time (protected):	• One hour Operation group meeting is held every Wednesday which is attended by operations group including PI and Co-Investigators to discuss weekly update<br>• One hour Data call every Tuesday to discuss data issues, progress of data collection, data management, analysis<br>• A one day bi- annual Investigators meeting to discuss about project updates, action items<br>• Six monthly site visits by Investigators	
	Funding:	NHLBI	
	Other:		
**Structures, Systems, Roles**	New Positions Developed:	We have leveraged several senior research associates affiliated with our partner institutions to assist in the capacity building activities. To date, these are unfunded positions.	
	Involvement of Ministry of Heath:	• Study team is working in close collaboration with Department of Health and Family Welfare, Punjab State and Ministry Health And Family Welfare, India (NCD Division) to implement project in Punjab<br>• Letter of Support from Ministry Health And Family Welfare, India (NCD Division) and Letter of Support from Civil Surgeon to facilitate data collection across intervention and control blocks<br>• Training of nurses and medical officers will occur in this calendar year.	

	Policy Development:	Dr Nikhil Tandon is a part of Expert group that is advising Policy makers in Ministry to Develop electronic case record form and Clinical decision Support System in the National program that is being rolled out Pan India for screening and Management of NCDs (*National Program For Control And Prevention Of Cancers, Diabetes, CVD And Stroke*.)	Our intervention is fully integrated within the GOI NCD portal.
	Forums, Dissemination, etc:	We plan to conduct a dissemination meeting.	All meetings have been conducted on zoom; local district meeting has been indefinitely postponed.
	Other:		
**Metrics**	IS Process Outcomes:	Guided by the RE-AIM framework, we will evaluate program **R**each (e.g., %'s screened in the community, tracked over time, and referred), **E**ffectiveness (e.g., % with controlled hypertension in the community), **A**doption (e.g., % of facilities using the I-TREC; provider reception to I-TREC), **I**mplementation outcomes (e.g., concordance between facility paper registry I-TREC database; routine operational costs per patient), and **M**aintenance (e.g., % of facilities utilizing I-TREC 6 months after the active intervention). Data will come from community-surveys, patient focus group discussions, provider interviews, and the I-TREC platform.	
	Clinical Outcomes:	Data points recorded in the I-TREC system will be analyzed by month to examine trends over time and seasonality. We will also evaluate change patient outcomes over time (e.g., mean SBP change). While several of the I-TREC platform indicators are purely descriptive measures of performance (e.g., mean time for data upload), other indicators of healthcare delivery may be compared between I-TREC facilities and comparison group facilities (e.g., patient volume).	
	Other:		

**Needs Assessment**	• Community epidemiological survey<br>• Qualitative interviews with community members and providers<br>• Facility assessments	While the baseline activities were conducted in person, midline survey activities have been delayed due to covid-19. We originally planned to shift to telephone only, then believed we could revert to in-person due to lower case loads and a vaccinated field staff. Then again, plans changed due to the current acute Covid-19 crisis.
**Additional Comments/Feedback on COVID-19 Impact Not Listed Above:**		

Kenya
TREIN/HyTREC Consortiumnd
Capacity Building Activities

The following table was designed to organize the different activities that each TREIN/HyTREC Study may be conducting in terms of capacity building. The Capacity Domains are based on the four dimensions of the Capacity Pyramid (Potter and Brough 2004) as incorporated into the Capacity Building Subcommittee mission statement: Tools, Skills, Staff/Infrastructure, and Structures/Systems/Roles. We have included some common activities or components of capacity building that may be a part of your study based on input from the Capacity Building Subcommittee members. Please feel free to add in additional activities that aren't listed here. Finally, we added sections on the metrics you will be using to evaluate your study and space to describe your needs assessment (if you are conducting one) to share with the Consortium. Please complete the table to the best of your ability and send questions to Annette Fitzpatrick (fitzpal@uw.edu) if needed. Thank you!

SITE	CAPACITY DOMAIN	Specific Activity/Components/Deliverables	Additional Details/Comment
	TOOLS	Equipment:	*Blood pressure monitors*
		Computers/Digital devices/Cell phones:	*Computers, Tablets, Laptops*
		Internet/Cell phone access:	Zoom, WI-FI router, cellphones
		IS Framework (if used):	PRECEDE-PROCEED framework
		Other:	
	SKILLS	Formal Training:	
		Informal Training:	Implementation Research Training<br>Basic research training<br>Human Centered Design training<br>Qualitative analysis software training
		Curriculum Used:	GACD implementation research training curriculum<br>Human centered design-ACUMEN Design Toolkit
		Other:	
	STAFF AND INFRASTRUCTURE	Mentors:	Constantine Akwanalo, Jemimah Kamano. Violet Naanyu, Rajesh Vendanthan, Sonak Pastakia, Thomas Valente, Ann Mwangi, Erick Finklestein, Tim Mercer, Gerry Bloomfield
		Investigator Time (protected):	Yes
		Funding:	NIH-NHLBI Funding

		Other:	
	**Structures, Systems, Roles**	New Positions Developed:	
		Involvement of Ministry of Heath:	Yes--the Kenyan Ministry of Health-Division of Non Communicable diseases from the National, County, Sub county and Health Facilities
		Policy Development:	Yes--New hypertension management guidelines developed in collaboration with stakeholder programs
		Forums, Dissemination, etc:	Stakeholders meetings<br>Conferences<br>Community entry meetings
		Other:	
	**Metrics**	IS Process Outcomes:	Acceptability and appropriateness testing.<br>Feasibility testing (pilot study)<br>Process evaluation by use of Saunders framework.
		Clinical Outcomes:	**<u>Primary outcome</u>**<br>One year absolute change in mean BP<br>**<u>Secondary outcomes</u>**<br>One-year change in overall CVD risk as measured by the QRISK2 score<br>One-year mortality rate<br>Hospital admissions<br>CVD complications<br>Change in CVD risk factors and behaviors<br>Medication adherence
		Other:	Process Outcomes<br>Referral Process Metrics<br>• Up-referral completion rate<br>• Down-referral completion rate<br>• Median referral completion time<br>Process Evaluation<br>• Fidelity (quality)<br>• Dose delivered (completeness)<br>• Dose received (exposure and satisfaction)<br>• Recruitment

			• Reach (participation rate)<br>• Context<br>Cost Effectiveness Outcomes<br>• Incremental cost effectiveness ratio
	**Needs Assessment**	Please describe:<br>Use mixed-methods approach, including: observational process mapping and gap assessment; baseline referral network analysis; and qualitative methods(Baraza-Community gatherings of 40 people and above, Focus group discussions and Key Informant Interviews) to identify facilitators, barriers, contextual factors, and readiness for change.	

Kenya
TREIN/HyTREC Consortium
Capacity Building Activities

Previously, you shared the below information regarding your capacity building activities. The Capacity Building Committee is summarizing this data in a manuscript; in order to ensure we have the most up-to-date information, we need your help.

Please review the table and: (1) update any incorrect/incomplete information and (2) indicate any changes or adaptations to these activities (e.g., delay in activity, changes in delivery mode, inability to complete) due to the COVID-19 pandemic. Please complete the table to the best of your ability and send questions to Mary Beth Weber (mbweber@emory.edu) if needed. This activity should take no more than 30 minutes to complete. We appreciate your assistance.

CAPACITY DOMAIN	Specific Activity/Components/Deliverables	Additional Details/Comment	Changes/Adaptations due to COVID-19
**TOOLS**	Equipment:	*Blood pressure monitors*	*The staff to ensure that they sanitize the machines before they use on the next participant*
	Computers/Digital devices/Cell phones:	*Computers, Tablets, Laptops*	*Adhere to COVID-19 measure i.e sanitizing*
	Internet/Cell phone access:	Zoom, WI-FI router, cellphones	Increased call activities to participants informing them of the delay in study activities.
	IS Framework (if used):	PRECEDE-PROCEED framework	No Change
	Other:		
**SKILLS**	Formal Training:		Virtual training of the staff on the current updated Protocol version 9.0 , study SOP for COVID-19, Good Documentation practices, Data Management and Consenting. This also includes the Manual of operations
	Informal Training:	Implementation Research Training<br>Basic research training<br>Human Centered Design training<br>Qualitative analysis software training	None
	Curriculum Used:	GACD implementation research training curriculum<br>Human centered design-ACUMEN Design Toolkit	The updated SOP's, Protocol and Manual of operations.
	Other:		
**STAFF AND INFRASTRUCTURE**	Mentors:	Constantine Akwanalo, Benson Njuguna, Jemimah Kamano. Violet Naanyu, Rajesh Vendanthan, Sonak	Constantine Akwanalo, Jemimah Kamano, Benson Njuguna, Violet Naanyu and Ann Mwangi

		Pastakia, Thomas Valente, Ann Mwangi, Erick Finklestein, Tim Mercer, Gerry Bloomfield	
	Investigator Time (protected):	Yes	Yes
	Funding:	NIH-NHLBI Funding	NIH-NHLBI Funding
	Other:		Support from other projects providing similar work within AMPATH research.
**Structures, Systems, Roles**	New Positions Developed:		None
	Involvement of Ministry of Heath:	Yes--the Kenyan Ministry of Health- Division of Non Communicable diseases from the National, County, Sub county and Health Facilities	No changes
	Policy Development:	Yes--New hypertension management guidelines developed in collaboration with stakeholder programs	None
	Forums, Dissemination, etc:	Stakeholders meetings<br>Conferences<br>Community entry meetings	None
	Other:		
**Metrics**	IS Process Outcomes:	Acceptability and appropriateness testing.<br>Feasibility testing (pilot study)<br>Process evaluation by use of Saunders framework.	None
	Clinical Outcomes:	**Primary outcome**<br>One year absolute change in mean BP<br>**Secondary outcomes**<br>One-year change in overall CVD risk as measured by the QRISK2 score<br>One-year mortality rate<br>Hospital admissions<br>CVD complications<br>Change in CVD risk factors and behaviors<br>Medication adherence	**None**

	Other:	Process Outcomes<br>Referral Process Metrics<br>• Up-referral completion rate<br>• Down-referral completion rate<br>• Median referral completion time<br>Process Evaluation<br>• Fidelity (quality)<br>• Dose delivered (completeness)<br>• Dose received (exposure and satisfaction)<br>• Recruitment<br>• Reach (participation rate)<br>• Context<br>Cost Effectiveness Outcomes<br>• Incremental cost effectiveness ratio	None
**Needs Assessment**	Please describe:<br>Use mixed-methods approach, including: observational process mapping and gap assessment; baseline referral network analysis; and qualitative methods(Baraza- Community gatherings of 40 people and above, Focus group discussions and Key Informant Interviews) to identify facilitators, barriers, contextual factors, and readiness for change.		This had already been completed before COVID-19
**Additional Comments/Feedback on COVID-19 Impact Not Listed Above:**			The project has been on hold during the period of March 2020 to March 2021. Thus no activities were being implemented apart from training of the staff.

Capacity building: Vietnam HY-TREC team

Study title: Đương đầu với bệnh Tăng huyết áp ở Việt Nam: Giải pháp từ Y tế cơ sở (Conquering Hypertension in Vietnam: Solutions at Grassroots level)

For health care workers:

We will implement the National Hypertension Control Program for both study groups (intervention and comparison groups). There will be a series of training sessions about hypertension prevention and management, which will be carried out at local district or provincial health departments. Research staff including physicians and nurses will participate in these trainings. Training sessions will be delivered in the collaboration with the Vietnam Ministry of Health.

There will be a series of training sections for Community health workers (CHWs) as well. CHWs will be taught simple techniques to help patients set goals for lifestyle changes, including salt and alcohol reduction, smoking cessation, and increased physical activity, and optimal medication adherence; they will also develop problem-solving strategies to achieve these goals. CHWs will be trained to instruct patients on how to use the blood pressure (BP) measurement devices to measure their BP at home and how to record and read their BP readings in the BP Log.

For researchers

Our multi-disciplinary and international team has a solid track record of working together across geographic distance with a foundation of mutual respect. This proposal offers the opportunity for continued capacity building. We have a Memorandum of Understanding in place between UMMS and Hanoi Medical University and the Hanoi School of Public Health for collaborating, teaching, and mentoring, offering opportunities to develop manuscripts and ancillary proposals. Although the Vietnam Health Strategy and Policy Institute has never applied for NIH funding, we worked closely scientifically and administratively in preparing and submitting this application. The UMass team will work with the Health Strategy and Policy Institute to enhance their ability to use advanced statistical techniques as presented in this proposal.

Vietnam
TREIN/HyTREC Consortium
Capacity Building Activities

Previously, you shared the below information regarding your capacity building activities. The Capacity Building Committee is summarizing this data in a manuscript; in order to ensure we have the most up-to-date information, we need your help.

Please review the table and: (1) update any incorrect/incomplete information and (2) indicate any changes or adaptations to these activities (e.g., delay in activity, changes in delivery mode, inability to complete) due to the COVID-19 pandemic. Please complete the table to the best of your ability and send questions to Mary Beth Weber (mbweber@emory.edu) if needed. This activity should take no more than 30 minutes to complete. We appreciate your assistance.

CAPACITY DOMAIN	Specific Activity/Components/Deliverables	Additional Details/Comment	Changes/Adaptations due to COVID-19
**TOOLS**	Equipment:<br>- DVD players<br>- Storytelling DVDs<br>- Blood pressure monitors	Health workers working at the study sites were provided DVD players, storytelling DVDs and blood pressure monitors as intervention and data collection tools. They were trained carefully of how to use these tools and to be able to guide patients.<br><br>The study patients were provided with DVD players, storytelling DVDs and blood pressure monitors as well. Local health workers instructed patients of how to use these tools to improve their blood pressure management at home.	
	Computers/Digital devices/Cell phones:<br>- Two laptops<br>- Two personal computers<br>- Five digital recorders<br>- One electric pointer<br>- One camera	These tools are provided for principal investigators and project staff for data collection, data analysis and management, and training for local health workers.	
	Internet/Cell phone access:		
	IS Framework (if used):		
	Other:		
**SKILLS**	Formal Training:<br>- Training sessions about hypertension prevention and management for community health workers (CHWs),	Two training courses were organized at the beginning and during the trial for 54 physicians and nurses, and 41 CHWs at study sites in September 2019 and July 2020.	

	physicians, and nurses at participating community health centers, delivered by the experts from the Vietnam National Heart Institute and the Vietnam Ministry of Health.<br>- Training sessions for CHWs about simple techniques to help patients set goals for lifestyle changes, including salt and alcohol reduction, smoking cessation, and increased physical activity, and optimal medication adherence. CHWs were trained to instruct patients on how to use the blood pressure (BP) measurement devices to measure their BP at home and how to record and read their BP readings in the BP Log.		
	- Training workshops about the study protocol and data collection activities needed for the baseline and follow-up surveys for researchers at the HSPI, delivered by study investigators	Several training workshops were organized for about ten researchers at HSPI	
	- The D&I Massive Open Online Course organized by the Capacity Building Sub-committee	One study staff has completed this course.	
	- Post graduate training	The project manager, Ms, Cuc Nguyen, has been admitted to the Ph.D. program in Health Management at the Hanoi Medical University. She is mentored by Dr. Hoa Nguyen, a key study investigator, and will use data from the hypertension trial for her dissertation.	

	Informal Training:	Administrative and project management training has been provided by the study investigators during the study period.	
	Curriculum Used:	The materials from the Vietnam National Hypertension Control Program was used in our training sessions about hypertension prevention and management.	
	Other:		
**STAFF AND INFRASTRUCTURE**	Mentors:	Dr. Hoa Nguyen is the mentor of Ms. Cuc Nguyen, the project manager, for her Ph.D. training mentioned in the previous section.	
	Investigator Time (protected):	~5%	
	Funding:	none	
	Other:		
**Structures, Systems, Roles**	New Positions Developed:	Several research assistant positions were created.	
	Involvement of Ministry of Heath:	Ministry of Health officials had participated in the training sessions mentioned in the previous section.	
	Policy Development:	NA	
	Forums, Dissemination, etc:	NA	
	Other:		
**Metrics**	IS Process Outcomes:	NA	
	Clinical Outcomes:	NA	
	Other:		
**Needs Assessment**	**Methodology:** During the first year of funding, the Health Strategy and Policy Institute conducted a Needs-Assessment to understand the study context and hypertension management related issues at the study sites. Data collection included key informant interviews (N=20) and focus group discussions (N=9). Respondents were the program coordinators, local health managers, community health workers, and patients with HTN.		We planned to conduct 3 rounds of the Needs Assessment study (in Years 1,3 and 5); however, due to COVID-19 pandemic, we were unable to conduct the second round in Year 3, we therefore will conduct the second round in Year 5.

	Following the key findings of need assessment conducted in the selected sites, the team has worked together to complement the proposed initially interventions, as follows:<br>• The study identified the knowledge gaps in the diagnosis, treatment, and management of non-communicable diseases among the primary care providers, including health staff at commune health centers and community health workers. Based on that, the necessary topics for training activities for health workers have been determined and used as the inputs for developing the training materials and curriculum.<br>• There were a number of logistic and organizational issues related to the training activities for health workers raised by local stakeholders. A training plan has been developed to integrate and complement the training activities conducted by the experts from the National Heart Institute and the Ministry of Health (eg. training contents, timelines, and trainers).<br>• Patients' expectations of better knowledge and skills in HTN-self management and control practices at their homes were carefully considered and discussed within the team. Several crucial topics have been determined and included in Storytelling DVDs that were developed as the core topics of "Storytelling" intervention.	
**Additional Comments/Feedback on COVID-19 Impact Not Listed Above:**		

Malawi
TREIN/HyTREC Consortiumnd
Capacity Building Activities

The following table was designed to organize the different activities that each TREIN/HyTREC Study may be conducting in terms of capacity building. The Capacity Domains are based on the four dimensions of the Capacity Pyramid (Potter and Brough 2004) as incorporated into the Capacity Building Subcommittee mission statement: Tools, Skills, Staff/Infrastructure, and Structures/Systems/Roles. We have included some common activities or components of capacity building that may be a part of your study based on input from the Capacity Building Subcommittee members. Please feel free to add in additional activities that aren't listed here. Finally, we added sections on the metrics you will be using to evaluate your study and space to describe your needs assessment (if you are conducting one) to share with the Consortium. Please complete the table to the best of your ability and send questions to Annette Fitzpatrick (fitzpal@uw.edu) if needed. Thank you!

CAPACITY DOMAIN	Specific Activity/Components/Deliverables	Additional Details/Comment
**TOOLS**	Equipment:	
	Computers/Digital devices/Cell phones/printers, photocopier and projector :	Each employee is required to have a laptop as well as a cell phone. The two items will enhance personal capacity for staff to deliver and achieve their project goals. This will further enhance their personal capacity in performing their day to day work.
	Internet/Cell phone access:	Availability of internet for employees and access to cell phones will help in every day dialogue between staff members such as communication with Principal Investigators, external consultants, government officials, partners and others. Without access to internet, there will be delayed communication which will result in delayed implementation of project activities. For instance, instead of employees travelling to one place for a meeting, they can have a skype call and this will save a lot of money on transport and accommodation as well as time. For instance, since the commencement of the project, several skype and phone meetings have been conducted.
	IS Framework (if used):	
	Other: vehicle	Availability of a vehicle is significant to the project to enhance institutional capacity as well as performance capacity. For instance, for employees to carry on field work in projected time, they require a vehicle as well as transport to meetings.
**SKILLS**	Formal Training:	Formal training to employees on the project is needed as it will give them confidence to perform properly. Therefore, there is need to identify what kind of formal training would participants require to have excellent performance. Participants need to be knowledgeable enough in their day to day work. This can be short term training as well long term training depending on flexibility of the project work load.
	Informal Training:	It is the duty of the project management to check if employees are deficient in some of the skills to perform their job to the best level of capacity. For instance, informal training can be allowing employees to learn from their mentors who have acquired those specific skills.

		Employees can also be supported and encourage to study on their own on specific topics about some of the skills.
	Curriculum Used:	Is a curriculum in place that matches with the skills, the project is aspiring its employees to achieve?
	Other: Short courses and trainings	Short courses and trainings act as incentives to the employees and therefore need to be included in the budget. Also they help to improve capacity institution if well done but if not properly done, they act as a waste of resources. For instance, if short courses or trainings are not relevant to the skills that are required to the implementation of the project.
**STAFF AND INFRASTRUCTURE**	Mentors:	
	Investigator Time (protected):	The Investigator time will enhance supervisory capacity to the project team. Does the investigator have enough time to make supervision for the project team? Are the reporting and monitoring tools in place to carry out project supervision?
	Funding:	Funding is one of the system's capacities that ensure that the project is running properly. For example, if the project has systems in place to ensure that all transactions are done according to the budget. These systems include managerial decisions such as hiring of new staff, procurement of project materials as well as well as proper filing and information systems.
	Other:	
**Structures, Systems, Roles**	New Positions Developed:	
	Involvement of Ministry of Heath:	The project has to make sure that inter-sectoral forums are put in place. The involvement of Ministry of Health ensures that the project is not pursuing its own interests. The involvement of Ministry of Health further allows that the health systems are strengthened making sure that good structures, systems and roles are in place.
	Policy Development:	Include policy makers by developing their skills to understand evidence based research that will help them in good decision making.
	Forums, Dissemination, etc:	Plan to engage communities in project planning for effective performance. Disseminate the project findings at intervals, i.e. interim findings as well as end of project findings.
	Other:	
**Metrics**	IS Process Outcomes:	
	Clinical Outcomes:	Identify the project clinical outcomes and plan how to address them.
	Other:	
**Needs Assessment**	Please describe:	

	Carry out a study to investigate needs assessment in the health facilities. This will help to help to address the holistic primary health care needs of individuals, families and communities. For example, the NCD BRITE project will carry out a needs assessment Phase 1 and 2 to investigate the health facility structures as well as capacity the health personnel has to deliver its services.

Malawi
TREIN/HyTREC Consortium
Capacity Building Activities

Previously, you shared the below information regarding your capacity building activities. The Capacity Building Committee is summarizing this data in a manuscript; in order to ensure we have the most up-to-date information, we need your help.

Please review the table and: (1) update any incorrect/incomplete information and (2) indicate any changes or adaptations to these activities (e.g., delay in activity, changes in delivery mode, inability to complete) due to the COVID-19 pandemic. Please complete the table to the best of your ability and send questions to Mary Beth Weber (mbweber@emory.edu) if needed. This activity should take no more than 30 minutes to complete. We appreciate your assistance.

CAPACITY DOMAIN	Specific Activity/Components/Deliverables	Additional Details/Comment	Changes/Adaptations due to COVID-19
**TOOLS**	Equipment:		
	Computers/Digital devices/Cell phones/printers, photocopier and projector :	Each employee is required to have a laptop as well as a cell phone. The two items will enhance personal capacity for staff to deliver and achieve their project goals. This will further enhance their personal capacity in performing their day to day work.<br><br>All employees the project have both a laptop as well as a cell-phone.	None
	Internet/Cell phone access:	Availability of internet for employees and access to cell phones will help in every day dialogue between staff members such as communication with Principal Investigators, external consultants, government officials, partners and others. Without access to internet, there will be delayed communication which will result in delayed implementation of project activities. For instance, instead of employees travelling to one place for a meeting, they can have a zoom call and this will save a lot of money on transport and accommodation as well as time. For instance, since the commencement of the	Internet continues to be provided to PI's and senior faculty to enable them to work especially when required to work remotely. During the first and second wave, mentored research updates, team meetings as well as workshops were being conducted via the zoom platform

		project, several zoom and phone meetings have been conducted.	
	IS Framework (if used):		None
	Other: vehicle	Availability of a vehicle is significant to the project to enhance institutional capacity as well as performance capacity. For instance, for employees to carry on field work in projected time, they require a vehicle as well as transport to meetings.	There have been no changes in the way that transport and transportation has been requested. Project staff continue to liaise with the University's transport office should the need arise.
**SKILLS**	Formal Training:	Formal training to employees on the project is needed as it will give them confidence to perform properly. Therefore, there is need to identify what kind of formal training would participants require to have excellent performance. Participants need to be knowledgeable enough in their day to day work. This can be short term training as well long term training depending on flexibility of the project work load.	Trainings attended have not been affected by the Covid-19 pandemic. Trainings have taken place via the zoom platform when participants have been unable to meet. During the months when the Covid-19 cases have dropped low enough to allow participants of trainings to meet in person all while still observing Covid-19 prevention measures.
	Informal Training:	It is the duty of the project management to check if employees are deficient in some of the skills to perform their job to the best level of capacity. For instance, informal training can be allowing employees to learn from their mentors who have acquired those specific skills. Employees can also be supported and encourage to study on their own on specific topics about some of the skills.	This has not been affected by the Covid-19 pandemic
	Curriculum Used:	Is a curriculum in place that matches with the skills, the project is aspiring its employees to achieve?	None
	Other: Short courses and trainings	Short courses and trainings act as incentives to the employees and therefore need to be included in the budget. Also they help to improve capacity institution if well done but if not properly done, they act as a waste of	Trainings attended by employees have not been affected by Covid-19 pandemic as they have been either online or have been attended when the number of Covid cases have been very low and

		resources. For instance, if short courses or trainings are not relevant to the skills that are required to the implementation of the project.	participants have observed Covid-19 sprevention measures.
**STAFF AND INFRASTRUCTURE**	Mentors:		
	Investigator Time (protected):	The Investigator time will enhance supervisory capacity to the project team. Does the investigator have enough time to make supervision for the project team? Are the reporting and monitoring tools in place to carry out project supervision?	The project lead has had adequate time to supervise the project activities and the project team.
	Funding:	Funding is one of the system's capacities that ensure that the project is running properly. For example, if the project has systems in place to ensure that all transactions are done according to the budget. These systems include managerial decisions such as hiring of new staff, procurement of project materials as well as well as proper filing and information systems.	There are been no adaptations in the way that the project or transactions are handled nor has there been a change in the way that administrative activities are done.
	Other:		None
**Structures, Systems, Roles**	New Positions Developed:		
	Involvement of Ministry of Heath:	The project has to make sure that inter-sectoral forums are put in place. The involvement of Ministry of Health ensures that the project is not pursuing its own interests. The involvement of Ministry of Health further allows that the health systems are strengthened making sure that good structures, systems and roles are in place.	The project continues to work with the Ministry of Health; officers of the Ministry of Health are frequently engaged in meetings and workshops organized by the project.
	Policy Development:	Include policy makers by developing their skills to understand evidence based research that will help them in good decision making.	None
	Forums, Dissemination, etc:	Plan to engage communities in project planning for effective performance.	The dissemination of needs assessment findings which was scheduled for the

		Disseminate the project findings at intervals, i.e. interim findings as well as end of project findings.	first quarter of 2021 has been changed. The needs assessments activities were affected by the closure of the college leading to a change in the timeline of needs assessment related activities.
	Other:		None
**Metrics**	IS Process Outcomes:		None
	Clinical Outcomes:	Identify the project clinical outcomes and plan how to address them.	None
	Other:		None
**Needs Assessment**	Please describe:<br>Carry out a study to investigate needs assessment in the health facilities. This will help to help to address the holistic primary health care needs of individuals, families and communities. For example, the NCD BRITE project will carry out a needs assessment Phase 1 and 2 to investigate the health facility structures as well as capacity the health personnel has to deliver its services.		The emergence of Covid-19 and the rise in cases led to the closure of the College of Medicine. Health centers also closed clinics and as such the needs assessment team could not conduct data collection at said facilities. Four facility structures were not visited due to these closures.<br>A manuscript was developed based off of the data that was collected before the shutting down of these facilities.
**Additional Comments/Feedback on COVID-19 Impact Not Listed Above:**<br>The project has trainees whose research projects it supports by way of mentorship, administrative support and the provision of small grants. Each individual project has its own timelines.			Some research projects were disrupted by the closing down of the College as well as the clinics. Timelines were affected and trainees resumed their normal project activities after the re-opening of clinics.

Nepal
TREIN/HyTREC Consortium
Capacity Building Activities

The following table was designed to organize the different activities that each TREIN/HyTREC Study may be conducting in terms of capacity building. The Capacity Domains are based on the four dimensions of the Capacity Pyramid (Potter and Brough 2004) as incorporated into the Capacity Building Subcommittee mission statement: Tools, Skills, Staff/Infrastructure, and Structures/Systems/Roles. We have included some common activities or components of capacity building that may be a part of your study based on input from the Capacity Building Subcommittee members. Please feel free to add in additional activities that aren't listed here. Finally, we added sections on the metrics you will be using to evaluate your study and space to describe your needs assessment (if you are conducting one) to share with the Consortium. Please complete the table to the best of your ability and send questions to Annette Fitzpatrick (fitzpal@uw.edu) if needed. Thank you!

SITE	CAPACITY DOMAIN	Specific Activity/Components/Deliverables	Additional Details/Comment
NEPAL	**TOOLS**	Equipment:	Office setup
		Computers/Digital devices/Cell phones:	Six laptops, one heavy printer, 2 cell phones with dialpad
		Internet/Cell phone access:	Continuous internet access provided by the institute
		IS Framework (if used):	
		Other:	
	**SKILLS**	Formal Training:	-Online courses<br>a. Fundamentals of Implementation Science<br>b. Leadership and Management in Health<br>c. Collaborative Institutional Training initiative<br><br>-Onsite courses/Workshops<br>a. Cardiovascular diseases research and future directions<br>b. Health Promotion and Behavior change<br>c. Quality Improvement in CVD<br>d. Qualitative Research and methods for CVD prevention and management<br>e. Fundamentals of Implementation Science<br>f. Biostatistics,1
		Informal Training:	-Other workshops and conferences
		Curriculum Used:	-Online course: Prepared by the host institute, University of Washington

			-Onsite courses: Prepared by the team of national co-investigator and international consultant
		Other:	-Pre and post evaluation for all the onsite courses based on the guideline of NIH
	**STAFF AND INFRASTRUCTURE**	Mentors:	National Mentors:<br>1. Prof. Rajendra Prasad Koju<br>2. Dr Archana Shrestha<br>3. Dr Biraj Man Karmacharya<br>4. Dr Rajeev Shrestha<br>5. Prof. Abhinav Vaidya.<br>6. Dr Natalia Oli<br>7. Dr Prajjwal Pyakurel<br>International mentors<br>1. Prof. Annette Fitzpatrick<br>2. Dr. David Citrin<br>3. Prof. Donna Spiegelman<br>4. Prof. Rifat Atun<br>5. Dr. Kenneth Sherr<br>6. Ms. Lori DiPrete Brown<br>7. Prof. Prabhakaran Dorairaj<br>8. Prof. Dong Xu (Roman)<br>9. Dr. Nona Sotoodehnia
		Investigator Time (protected):	-Principal Investigator (3.6 calendar months)<br>-Co-Directors (7.2 calendar months)<br>-Co-investigators (2.4 calendar months)
		Funding:	National Heart, Lung, and Blood Institute (NHLBI)
		Other:	-Project Coordinator, project officers (2), research officer, research assistants (2)
	**Structures, Systems, Roles**	New Positions Developed:	Sixteen research fellows
		Involvement of Ministry of Heath:	-MoU with apex body of the Government of Nepal for health related researches, Nepal Health Research Council<br>-Members in steering committee, advisory committee, needs assessment task force, research team<br>-Planning to involve as research fellows
		Policy Development:	-N/A
		Forums, Dissemination, etc:	-Health journalist appointed as one of the member of steering committee.<br>-Newsletter and news blurb in the hospital website.<br>-International Annual symposium at Kathmandu

			-Poster presentation in the symposium<br>-International Conferences, CUGH<br>-NHRC 5th Summit<br>-Article publications in Journal
		Other:	
	**Metrics**	IS Process Outcomes:	-Collaborations with international and regional partners.<br>-Consolidation of members in different teams<br>-Achievement of deliverables by the research fellows.<br>-Development of atleast one translational research plan.
		Clinical Outcomes:	
		Other:	Policies briefs for addressing the gap in the health system assessment.
	**Needs Assessment**	Please describe: Protocol	

Nepal
TREIN/HyTREC Consortium
Capacity Building Activities

Previously, you shared the below information regarding your capacity building activities. The Capacity Building Committee is summarizing this data in a manuscript; in order to ensure we have the most up-to-date information; we need your help.

Please review the table and: (1) update any incorrect/incomplete information and (2) indicate any changes or adaptations to these activities (e.g., delay in activity, changes in delivery mode, inability to complete) due to the COVID-19 pandemic. Please complete the table to the best of your ability and send questions to Mary Beth Weber (mbweber@emory.edu) if needed. This activity should take no more than 30 minutes to complete. We appreciate your assistance.

CAPACITY DOMAIN	Specific Activity/Components/ Deliverables	Additional Details/Comment	Changes/Adaptations due to COVID-19
**TOOLS**	Equipment:	Office setup, one heavy printer (3 in one), tape recorders, foot pedals for transcribing data	We worked from home during lockdown due to COVID. When we visited office, we had meetings with appropriate precautionary measures mandated by the Nepalese government.
	Computers/Digital devices/Cell phones:	Six laptops, 7 cell phones with dialpad	We conducted telephone interviews with our respondents.
	Internet/Cell phone access:	-Continuous internet access provided by the institute at office,<br>-Telephone recharged by the institute	We communicated via telephone and online meetings most of the time due to lockdown due to COVID.
	IS Framework (if used):		
	Other:		
**SKILLS**	Formal Training:	-Online courses<br>a. Fundamentals of Implementation Science<br>b. Leadership and Management in Health<br>c. Collaborative Institutional Training initiative<br>d. Economic Evaluation in Global health<br>e. WHO TDR Massive Open Online Course (MOOC) on Implementation Research<br>f. Policy Development and Advocacy for Global Health<br>g. Introduction to Epidemiology for Global Health<br>h. Project Management in Global Health<br>i. Systematic Review and Meta-Analysis	-We completed major training onsite before COVID pandemic.<br>During lockdown due to COVID pandemic we could not conduct onsite training for our fellows. Thus, we hosted onsite courses from the University of Washington.

		-Onsite courses/Workshops<br>a. Cardiovascular diseases research and future directions<br>b. Health Promotion and Behavior change<br>c. Quality Improvement in CVD<br>d. Qualitative methods and data analysis for CVD prevention and management<br>e. Advanced Implementation Science<br>f. Biostatistics,I,II,III and IV<br>g. Health Policy workshop<br>h. Grant Writing workshop	
	Informal Training:	-Continuous mentorship and guidance by national and international mentors<br>-Manuscript writing workshops (frequent)<br>-Coding and data analysis workshop<br>-Poster preparation and presentation training	
	Curriculum Used:	-Online course:<br>Prepared by the host institute and subject expertise, University of Washington<br>-Onsite courses: Prepared by the team of national co-investigator and international consultant	
	Other:	-Pre and post evaluation for all the onsite courses based on the guideline of NIH<br>-GACD Implementation Science Research Training School<br>-Our fellows secured various international training and fellowship programs using the professional network of this fellowship.<br>a. The Bernard Lown Scholars in Cardiovascular Health Program<br>b. Global Health Delivery Intensive Program offered by Harvard University.	Due to COVID, each fellow completed their international training online and were not able to visit USA for training.
**STAFF AND INFRASTRUC TURE**	Mentors:	National Mentors:<br>1. Prof. Rajendra Prasad Koju<br>2. Dr Archana Shrestha<br>3. Dr Biraj Man Karmacharya<br>4. Dr Rajeev Shrestha	

		5. Prof. Abhinav Vaidya.<br>6. Dr Natalia Oli<br>7. Dr Prajjwal Pyakurel<br><br>International mentors<br>1. Prof. Annette Fitzpatrick<br>2. Dr. David Citrin<br>3. Prof. Donna Spiegelman<br>4. Ms. Lori DiPrete Brown<br>5. Prof. Prabhakaran Dorairaj<br>6. Prof. Dong Xu (Roman)<br>7. Dr. Nona Sotoodehnia	
	Investigator Time (protected):	-Principal Investigator (3.6 calendar months for four years)<br>-Co-Directors (7.2 calendar months year year 1 thru 4)<br>-Co-investigators (2.4 calendar months year 1,4; 1.2 calendar months year 2 and 3)	
	Funding:	National Heart, Lung, and Blood Institute (NHLBI)	
	Other:	-Research Project Coordinator (1), project officers (3), research assistants (2)	
**Structures, Systems, Roles**	New Positions Developed:	-Twenty three research fellows	
	Involvement of Ministry of Health and Population, apex bodies working for CVDs in Nepal, civil society organisations for patients with CVD	-MoU with the apex body of the Government of Nepal for health related researches, Nepal Health Research Council<br>-Members in steering committee, advisory committee, needs assessment task force, research team<br>-Recruited seven government employees as research fellows<br>-Regular communication via online as well as physical meetings	Online meeting via google meet.
	Policy Development:	-N/A	
	Forums, Dissemination, etc:	-A health journalist appointed as one of the members of the steering committee.	-Post COVID pandemic, we held online meetings and communicated by telephone when required.<br>-We are also not able to hold an NHLBI site visit.

		-Newsletters and news blurb on the hospital website.<br>-International Annual symposium at Kathmandu<br>-Poster presentation in the symposium<br>-International Conferences, CUGH<br>-5th, 6th and 7th national summit of health and population scientists in Nepal<br>-Article publications in Journal<br>-Second Stakeholders' Meeting<br>-Implementation Science Workshop (facilitated by fellows)<br>-Nepalese Society of Community Medicine (Nescom) Conference<br>-Online Biannual TREIN/ Hy-TREC meeting of Ghana and upcoming GRIT cc meeting<br>-Sustaining Global Capacity for Implementation Research for Health in Low- and Middle-Income Countries	-Due to COVID pandemic, our fellows presented their poster and presentation online
	Other:	-Early Stage Investigator award<br>-Two fellows secured PhD and one fellows started her post doctoral training	
**Metrics**	IS Process Outcomes:	N/A	
	Clinical Outcomes:	N/A	
	Other:	-Certificate of course completion with credit points after each course<br>-Collaborations with international and regional partners.<br>-Consolidation of members in different teams<br>-Achievement of deliverables by the research fellows.<br>-Development of three implementation science research proposals and their execution by fellows.<br>-Policy briefs for addressing the gap in the health system assessment.	

**Needs Assessment**	Please describe:<br>-Based on the World Health Organization's health system building block framework we developed a needs assessment protocol adopting USAIDs Health System assessment manual 2.0.<br>-We analyzed data of cardiovascular disease burden and stroke from IHME, Global Burden of Disease data site.<br>-One on burden of CVD is available here.<br>- One paper has been accepted in BMC Public Health<br>- Seven other papers are in review	-We completed activities of needs assessment before the COVID pandemic hit Nepal.
**Additional Comments/Feedback on COVID-19 Impact Not Listed Above:**		-We planned to perform a rapid assessment of infrastructure and readiness of Nepalese health system. Due to COVID pandemic we halted the assessment. -We changed the modality of data collection for our qualitative perception study. Initially we planned for face to face interviews, later we conducted interviews via telephone after receiving approval from NHRC.

Rwanda
TREIN/HyTREC Consortium
Capacity Building Activities

The following table was designed to organize the different activities that each TREIN/HyTREC Study may be conducting in terms of capacity building. The Capacity Domains are based on the four dimensions of the Capacity Pyramid (Potter and Brough 2004) as incorporated into the Capacity Building Subcommittee mission statement: Tools, Skills, Staff/Infrastructure, and Structures/Systems/Roles. We have included some common activities or components of capacity building that may be a part of your study based on input from the Capacity Building Subcommittee members. Please feel free to add in additional activities that aren't listed here. Finally, we added sections on the metrics you will be using to evaluate your study and space to describe your needs assessment (if you are conducting one) to share with the Consortium. Please complete the table to the best of your ability and send questions to Annette Fitzpatrick (fitzpal@uw.edu) if needed. Thank you!

SITE	CAPACITY DOMAIN	Specific Activity/Components/ Deliverables	Additional Details/Comment
	**TOOLS**	Equipment:	*Nil*
		Computers/Digital devices/Cell phones:	*Nil*
		Internet/Cell phone access:	Nil
		IS Framework (if used):	Nil
		Other:	
	**SKILLS**	Formal Training:	*Our U24 program provides a unique opportunity to train dynamic trans-disciplinary Rwandan scientific and public health teams to design and engage in T4TR for the control of hypertension in Rwanda. our program aims to train scientists to provide them with competencies required to enhance uptake of proven interventions for control of hypertension. The main aims of this program are to design training activities to build capacity for T4TR to improve prevention and control of hypertension, to conduct needs assessment on facilities, resources, skills and data to advance T4TR capacity for sustainable hypertension control, and to develop a comprehensive context-specific hypertension research plan. Formal training efforts are geared at implementing skills training through in-country workshops, distance learning/training, cyber-seminars and mentorships to develop competencies for dissemination and implementation research (D&IR). The future long-term goal is that successful trainees apply for established Masters or PhD level training in D&IR*

		Informal Training:	
		Curriculum Used:	*A majority of public health practitioners in Rwanda are in some way involved in dissemination and implementation science for control of hypertension, with little or no prior training. During our D&I training, we have endeavored to use trainers with extensive D&I knowledge and experience who bring on board curriculum that put forward many innovative and evidence based practices in D&I*
		Other:	
	**STAFF AND INFRASTRUCTURE**	Mentors:	*As part of our program we are keen to develop long lasting, purposeful and strategic mentorship- trainee relationships between experienced researchers, public health practitioners, faculty in universities and other more experience peers for more positive and more long term outcomes, academic progress, increased productivity in both research, public health and academia for the trainees in Rwanda*
		Investigator Time (protected):	*Nil*
		Funding:	*Although we don't provide direct funding as part of capacity building of our program trainees, we train, support and encourage trainees to look for alternative funding opportunities as part of our sustainability model. The aim is to enable Rwandan scholars to successfully compete for research grant funding on dissemination and implementation (D&I) studies for HTN control in Rwanda.*
		Other:	
	**Structures, Systems, Roles**	New Positions Developed:	*Nil*
		Involvement of Ministry of Heath:	*We are developing multi-disciplinary teams and solidifying collaborations and networks for T4TR capacity in Rwanda. We will train Rwandan scientists and other public health providers to gain T4TR competencies and establish connections with community stakeholders to identify their needs for HTN/CVD control. This will complement the Government of Rwanda's mission to prevent epidemiological transition to non-communicable diseases (NCDs)*
		Policy Development:	*In working with different pertinent stakeholders to provide updated and valid information on a relatively new field of dissemination and implementation or late stage translational research for control of hypertension in Rwanda, the program outputs will be disseminated and shared with high level stakeholders to form part of a critical message to create evidence-based policies at ministerial levels and also compliment other government and partner efforts to combat chronic illnesses in Rwanda*

		Forums,<br>Dissemination, etc:	*Our program will carry out an active process to communicate results of the needs assessment and skills development to Rwandan communities and end users. The goal is to obtain feedback from stakeholders and develop a context-specific plan and utilize well-validated data for knowledge utilization in Rwanda and other LMICs. The dissemination will target social and cultural Rwandan perspectives in terms of behavioral/habitual activities that strongly influence HTN/CVD related morbidities. We will package the messages for a particular target audience based on demographic characteristics such as age, gender, social classes and other factors. Dissemination strategies will include: linkage and exchange events to share relevant HTN research syntheses; developing a user driven dissemination strategy through various media engagements; developing researcher/knowledge user networks especially community health workers (CHW). This particular group will form intermediaries to facilitate knowledge utilization and form a strong bridge between researchers and other knowledge users. These are critical elements for HTN translation; public health awareness and control to support evidence to shape decisions interpret, and adapt evidence to the local Rwandan context*
		Other:	
	**Metrics**	IS Process Outcomes:	1. *Number of trainees trained by the program*<br>2. *Evaluation of D&IR needs assessment for control of hypertension in Rwanda*<br>3. *Creation of a context specific research strategy*
		Clinical Outcomes:	*Nil*
		Other:	
	**Needs Assessment**	Please describe:<br><br>*We will conduct a need assessment and prioritize country-specific interventions for HTN; the primary risk factor for cardiovascular diseases (CVD). We will assess needs to support development of a context-specific research plan for T4TR capacity. Lack of infrastructure and staff capacity in Rwanda constrains knowledge dissemination and evidence-based behavioral change interventions for individuals with and/or at risk for HTN/CVD. This increases the gap between the best evidence on lifestyle behavioral interventions, clinical care and prevention efforts.*	

Rwanda
TREIN/HyTREC Consortium
Capacity Building Activities

Previously, you shared the below information regarding your capacity building activities. The Capacity Building Committee is summarizing this data in a manuscript; in order to ensure we have the most up-to-date information, we need your help.

Please review the table and: (1) update any incorrect/incomplete information and (2) indicate any changes or adaptations to these activities (e.g., delay in activity, changes in delivery mode, inability to complete) due to the COVID-19 pandemic. Please complete the table to the best of your ability and send questions to Mary Beth Weber (mbweber@emory.edu) if needed. This activity should take no more than 30 minutes to complete. We appreciate your assistance.

CAPACITY DOMAIN	Specific Activity/Components/Deliverables	Additional Details/Comment	Changes/Adaptations due to COVID-19
	Equipment:	*Nil*	*Due to COVID-19 most of the work was done via computers and cell phones in digital communication.*
	Computers/Digital devices/Cell phones:	*Nil*	
	Internet/Cell phone access:	Nil	We updated internet connectivity to broadband internet connectivity for full-time staff using 4G
	IS Framework (if used):	Nil	
	Other:		
**SKILLS**	Formal Training:	*Our U24 program provides a unique opportunity to train dynamic trans-disciplinary Rwandan scientific and public health teams to design and engage in T4TR for the control of hypertension in Rwanda. our program aims to train scientists to provide them with competencies required to enhance uptake of proven interventions for control of hypertension. The main aims of this program are to design training activities to build capacity for T4TR to improve prevention and control of hypertension, to conduct needs assessment on facilities, resources, skills and data to advance T4TR capacity for sustainable hypertension control, and to*	*During different phases of lockdown due to COVID-19, most of the training activities that were planned were done virtually.*<br><br>• ***Grant writing skills training** for hypertension control was delivered during **4th Virtual Annual NCDs, Hypertension, and implementation Science Symposium**.*<br><br>• *Two-weeks scientific symposium to develop trainees' capacity in dissemination and implementation (D&I) research for writing successful proposals and Manuscripts;*<br><br>• *We conducted the workshop to understand Trainees proposals on HTN control in Rwanda;*

		develop a comprehensive context-specific hypertension research plan. Formal training efforts are geared at implementing skills training through in-country workshops, distance learning/training, cyber-seminars and mentorships to develop competencies for dissemination and implementation research (D&IR). The future long-term goal is that successful trainees apply for established Masters or PhD level training in D&IR	• *We conducted community awareness educational campaigns using social media, radio and TV in partnership with Rwanda Heart Foundation, Rwanda NCD Alliance (after COVID-19 lockdown was eased, we leveraged these activities through social distancing physical activities/ exercise) campaigns called 'Use your Heart to beat CVDs"*
	Informal Training:		*We conduct weekly virtual call for publication planning and implementation. During this call, mentors help trainees on abstract and manuscript writing, publication skills.*<br><br>*We also conduct weekly call with our counterparts in Washington St Louis where we discuss grants applications, publication progress and presentation skills by junior trainees.*
	Curriculum Used:	*A majority of public health practitioners in Rwanda are in some way involved in dissemination and implementation science for control of hypertension, with little or no prior training. During our D&I training, we have endeavored to use trainers with extensive D&I knowledge and experience who bring on board curriculum that put forward many innovative and evidence based practices in D&I*	*Despite COVID-19, experts in D&I on hypertension interact virtually with trainers in Rwanda and they keep using the curriculum that would have been used in face-to-face mode.*
	Other:		
**STAFF AND INFRASTRUCTURE**	Mentors:	*As part of our program we are keen to develop long lasting, purposeful and strategic mentorship- trainee relationships between experienced researchers, public health practitioners, faculty in universities and other more*	*With COVID-19, mentors engage mentees via phone calls, emails and weekly virtual meetings*

		experience peers for more positive and more long term outcomes, academic progress, increased productivity in both research, public health and academia for the trainees in Rwanda	
	Investigator Time (protected):	*Nil*	
	Funding:	*Although we don't provide direct funding as part of capacity building of our program trainees, we train, support and encourage trainees to look for alternative funding opportunities as part of our sustainability model. The aim is to enable Rwandan scholars to successfully compete for research grant funding on dissemination and implementation (D&I) studies for HTN control in Rwanda.*	*Currently, we have provided Grant writing training to Rwandan scholars. Six (6) of the trainees secured PhD position grants and others are nearly completing their grants applications.*
	Other:		
**Structures, Systems, Roles**	New Positions Developed:	*Nil*	
	Involvement of Ministry of Heath:	*We are developing multi-disciplinary teams and solidifying collaborations and networks for T4TR capacity in Rwanda. We will train Rwandan scientists and other public health providers to gain T4TR competencies and establish connections with community stakeholders to identify their needs for HTN/CVD control. This will complement the Government of Rwanda's mission to prevent epidemiological transition to non-communicable diseases (NCDs)*	*Among the trainees we have been able to retain in the capacity building program are 2 hospitals directors and one director of Cardiovascular diseases in Rwanda Biomedical Center. These health care leaders represent the Ministry of Health in the districts they operate in.*
	Policy Development:	*In working with different pertinent stakeholders to provide updated and valid information on a relatively new field of dissemination and implementation or late stage translational research for control of hypertension in Rwanda, the program*	*After the needs assessment conducted in 3 district hospitals, recommendations about decentralization of NCD services to community and health centers have been adopted which is a proof that the Ministry of Health considers our work beneficial to the entire heath sector.*

		outputs will be disseminated and shared with high level stakeholders to form part of a critical message to create evidence-based policies at ministerial levels and also compliment other government and partner efforts to combat chronic illnesses in Rwanda	
	Forums, Dissemination, etc:	*Our program will carry out an active process to communicate results of the needs assessment and skills development to Rwandan communities and end users. The goal is to obtain feedback from stakeholders and develop a context-specific plan and utilize well-validated data for knowledge utilization in Rwanda and other LMICs. The dissemination will target social and cultural Rwandan perspectives in terms of behavioral/habitual activities that strongly influence HTN/CVD related morbidities. We will package the messages for a particular target audience based on demographic characteristics such as age, gender, social classes and other factors. Dissemination strategies will include: linkage and exchange events to share relevant HTN research syntheses; developing a user driven dissemination strategy through various media engagements; developing researcher/knowledge user networks especially community health workers (CHW). This particular group will form intermediaries to facilitate knowledge utilization and form a strong bridge between researchers and other knowledge users. These are critical elements for HTN translation; public health awareness and control to support evidence to shape decisions interpret, and adapt evidence to the local*	*Due to COVID-19, community awareness was only done via Radio and television.*<br>*The face to face sessions that were planned did not take place.*

		Rwandan context	
	Other:		
**Metrics**	IS Process Outcomes:	4. *Number of trainees trained by the program*<br>5. *Evaluation of D&IR needs assessment for control of hypertension in Rwanda*<br>6. *Creation of a context specific research strategy*	1. *25 trainees*<br>2. *3 district hospitals have been assessed.* ***Participants: 223 health care providers (MD, Nurses, Midwives)***<br>3. *We are working with the Ministry of health to increase uptake of EBIs from the community level to tertiary care.*
	Clinical Outcomes:	*Nil*	
	Other:		
**Needs Assessment**	Please describe:<br><br>*We will conduct a need assessment and prioritize country-specific interventions for HTN; the primary risk factor for cardiovascular diseases (CVD). We will assess needs to support development of a context-specific research plan for T4TR capacity. Lack of infrastructure and staff capacity in Rwanda constrains knowledge dissemination and evidence-based behavioral change interventions for individuals with and/or at risk for HTN/CVD. This increases the gap between the best evidence on lifestyle behavioral interventions, clinical care and prevention efforts.*		
**Additional Comments/Feedback on COVID-19 Impact Not Listed Above:**			
