# Supplementary material for: Global implementation research capacity building to address cardiovascular disease: An assessment of efforts in eight countries
Source: PLOS Glob Public Health. 2023 Sep 14;3(9):e0002237. doi: 10.1371/journal.pgph.0002237 (PMC10501667; doi:10.1371/journal.pgph.0002237)
Supplement: Attachment — Submitted filename: Capacity Paper response to review comments.docx [file pgph.0002237.s004.docx]

Response to Reviewer Comments

Thank you to the reviewers and editors for their complements and helpful suggestions. We have addressed the suggestions as follows (reviewer comments shown below in *italics*; the marked manuscript shows changes highlighted in yellow).

**Editorial Comments:**

*Please include a complete copy of PLOS’ questionnaire on inclusivity in global research in your revised manuscript. Our policy for research in this area aims to improve transparency in the reporting of research performed outside of researchers’ own country or community. The policy applies to researchers who have travelled to a different country to conduct research, research with Indigenous populations or their lands, and research on cultural artefacts. The questionnaire can also be requested at the journal’s discretion for any other submissions, even if these conditions are not met.  Please find more information on the policy and a link to download a blank copy of the questionnaire here:* [*https://journals*](https://journals/)*.plos.org/plosone/s/best-practices-in-research-reporting. Please upload a completed version of your questionnaire as Supporting Information when you resubmit your manuscript.*

The PLOS’ questionnaire on inclusivity in global research is included with the resubmission.

*Please provide separate figure files in .tif or .eps format only and remove any figures embedded in your manuscript file. Please also ensure all files are under our size limit of 10MB.*

The figure has been removed from the manuscript and is uploaded as a .tif file. We ran the figure through the PACE software, and the uploaded version has been corrected to fit publication specifications.

**Reviewer Comments**

**Reviewer 1:**

1. *The research question for the study is not adequately substantiated. The introduction section describes the benefit of the study as a learning experience for researchers, public health practitioners and clinicians. However, the manuscript did not identify this as a knowledge gap that warrants further research.*

We have added text to the introduction section to provide additional support for this analysis as follow (lines 143-149):

“To date, few studies provide detailed reporting of capacity building activities, and the GRIT consortium is a unique opportunity to present a descriptive analysis of the types of capacity building activities across multiple LMIC settings. Characterization of capacity building activities in LMICs, using frameworks like the one developed by Potter and Brough, is an important contribution to the literature and for practitioners. These data provide key details on the types of activities being implemented in real-world capacity building efforts and offer considerations for researchers and implementers.”

2. *The study provides detail description of project activities from the GRIT sites. However these represent superficial description of the phenomenon lacking sufficient depth and technical rigour.*

This manuscript presents a global descriptive analysis, with the technical rigor of having used a systemic capacity building model on independently planned studies. Despite this, the strategies used to improve capacity building were quite similar across sites. We have included citations for methods for the sites when available (please see Table 1) where the reader can find additional details on the intervention or program design. We have added a note directing the reader to these resources (lines 162-163: “additional program/intervention details can be found in the project-specific methods publications.” Furthermore, we agree that the method used to collect the data could have resulted in data that is lacking the nuance that might have been identified in a more in-depth analysis (e.g., qualitative interviewing of multiple team members). We expanded on our discussion of this limitation to note this (lines 512-513: “Future studies of this type should consider mixed methods data collection to explore capacity building activities and their impacts.”).

3. *The study employed Potter and Brough model for analysis of the data. However, detail description of the analysis process is lacking.*

We have added additional information to the analysis paragraph to provide more details on the analysis process. The paragraph now reads (lines 199-213):

“Members of the manuscript writing team (MBW, AAB, AR, AF, MPF) conducted a thematic review of the information provided in the data collection tool. Each of the five reviewers reviewed one category of data or the needs assessment; data were not double coded. Each reviewer pulled the data pertaining to their assigned category from the data collection tool, documented commonalities and differences across country sites, then created summary documents and tables including both quantitative (e.g., number of sites reporting various items) and qualitative (e.g., descriptions of needs assessments used) outcomes for each of the four tiers of the systemic capacity building model as well as a summary of each project’s needs assessment activities, program/study metrics, and COVID-19 adaptations. Stakeholder checking was conducted by sending tables and supporting text to the sites’ PIs and members of the Capacity Building Subcommittee who added additional detail and clarification. Adaptations to the capacity building activities due to COVID-19 were similarly reviewed and summarized. Additional updates were added upon review of the manuscript by members of the Capacity Building Subcommittee to reflect additional changes during the COVID-19 pandemic in April 2022 not captured by the updated data collection tool.”

4. *The discussion section repeats the results from the study. Analysis of the results against previous work and the contribution of the study to fill or modify current knowledge in the field is not adequately described.*

Our discussion section uses the following format to guide it: (1) a summary of the findings according the framework used (lines 371-397, 458-470, and 476-490); (2) comparisons of our findings with other literature (lines 398-247, 470-475, 490-497); (3) a discussion of additional places were growth is needed in capacity building in LMICs (lines 428-457); and (4) strengths and weaknesses (lines 498-513). We have added additional text to the discussion of strengths to highlight how this fills important gaps in the literature.

5. *There are some typos grammatical errors that require correction (e.g. line 103, 'tier', not 'tiers'; line 141 'includes', not 'including'*

Thank you for bring these to our attention. We have made the appropriate changes.

6. *Abbreviations need to be written in full when introduced for the first time (e.g. line 149 'IS process...')*

Thank you for catching that oversight. We checked the other abbreviations to ensure they are spelled out.

7. *The study used data from different studies conducted in the sites. It is not clear if ethics approval of these individual studies offered use of the data for secondary analysis such as for this study.*

This analysis did not include any participant-level data, so consent was not needed. As described in the Ethics Approval Section: “This descriptive study evaluates program-level data and is not human research; review board approval was not needed. The analysis reported in this manuscript did not include any patients or the public, collecting data only from the project teams through the PIs.”

8. *The study mentions the activities as easier and harder to implement. What is the criteria to categorise project activities as easier or harder to implement? Similarly 'formal' and 'informal' training requires definition.*

The Potter and Brough model uses the terms easier and harder to implement to indicate items that are technical in nature (e.g., purchasing tools) and sociocultural in nature (e.g., changing systems). Formal and informal training are defined as follows: Formal training is planned and structured training including courses, lectures, training classes; and informal training is unplanned/unstructured training including mentoring activities.

We updated the text to clarify these issues.

9. *In table 2, do empty cells meant the activity is not performed in the corresponding site? An X and tick symbols could provide better readability.*

As suggested, we added to check marks and -’s to more clearly show which items were performed/used or not performed/used, respectively, at each site.

10. *The study includes some subjective conclusions such as 'good model' or 'proven' frameworks. What measures did the authors use to report the GRIT model as a good model? What criteria was used to describe the frameworks as proven frameworks?*

We agree that this manuscript lacks the outcomes assessment to say that GRIT is a good model. We have softened that language (e.g., “may be a good model”). Furthermore, we have softened the language around the frameworks, using instead “common implementation science frameworks.”

11. *While the projects have made adaptations following COVID, it is not clear what the impact of such adaptations were on the outcomes of capacity building activities.*

Although we did not directly evaluate the outcomes associated with COVID-related changes, the project teams reported that these adaptations allowed the projects to continue on modified timelines. We added language to reflect this in the results and discussion sections.

12. *Some of the arguments described in the discussion section lack citation (e.g. the first sentence in paragraph two, page 20; the sentence in line 395 page 22; the sentence in line 402 page 22).*

We apologize for the lack of clarity with these citations. In these cases, the citation can be found at the end of the discussion of that reference. We have added the citation to each sentence referencing those manuscripts. Sentences without citations reflect the thoughts of the authors of this manuscript.

13. *Evaluation of the capacity building activities was not conducted and this has been indicated as a limitation. However, the method section describes evaluation of the activities.*

We have updated the analysis section title to “Reporting and Categorization of Capacity-Building Activities” to better reflect our activities.

**Reviewer 2:**

14. *It will be great to have some background descriptions on the social, economic and political factors that influence the hypertension management in the countries, which will be helpful to understand the context.*

The goal of this manuscript is to provide a high-level view of the capacity building activities across the GRIT network. We agree, however, that providing some background information on the challenges to implementation in the countries included in this network is important. We have added several paragraphs on challenges to the background section. A more thorough discussion of specific environmental and setting specific challenges in each country can be found in the cited methods papers for each study/program.

15. *In Table 3, better to specify the adaptions to capacity building activities by each country, since each country might have different scenario.*

This manuscript reports on a global descriptive analysis, which sought to explore differences and commonalities of capacity building activities across sites with a lesser focus on site-specific activities. The strategies, both pre-COVID and in response to COVID, were quite similar across sites and any differences were noted in the text. We added a comment to the discussion to clarify this: (lines 486-490: “Overall, COVID-related adaptations were similar across GRIT projects, with observed differences based on variation in planned program or intervention activities not location-specific barriers.”)

16. *In Discussion, it will be better to include suggestions to improve capacity building skills in different countries. This will provide insights for further program conduction in these countries.*

This data is or will be available in the publications from individual GRIT projects and will include evaluations of the program and intervention components. In this manuscript, we focused on the global-level description of capacity building activities.
